# Pulsed Laser-Assisted Helium Ion Nanomachining of Monolayer Graphene—Direct-Write Kirigami Patterns

**DOI:** 10.3390/nano9101394

**Published:** 2019-09-30

**Authors:** Cheng Zhang, Ondrej Dyck, David A. Garfinkel, Michael G. Stanford, Alex A. Belianinov, Jason D. Fowlkes, Stephen Jesse, Philip D. Rack

**Affiliations:** 1 Department of Materials Science and Engineering, University of Tennessee, Knoxville, TN 37996, USA; czhang68@utk.edu (C.Z.); dgarfink@vols.utk.edu (D.A.G.); mstanfo3@gmail.com (M.G.S.); 2 Center for Nanophase Materials Sciences, Oak Ridge National Laboratory, Oak Ridge, TN 37831, USA; dyckoe@ornl.gov (O.D.); belianinova@ornl.gov (A.A.B.); fowlkesjd@ornl.gov (J.D.F.); sjesse@ornl.gov (S.J.)

**Keywords:** graphene, direct-write kirigami, nanopatterning, pulsed laser

## Abstract

A helium gas field ion source has been demonstrated to be capable of realizing higher milling resolution relative to liquid gallium ion sources. One drawback, however, is that the helium ion mass is prohibitively low for reasonable sputtering rates of bulk materials, requiring a dosage that may lead to significant subsurface damage. Manipulation of suspended graphene is, therefore, a logical application for He^+^ milling. We demonstrate that competitive ion beam-induced deposition from residual carbonaceous contamination can be thermally mitigated via a pulsed laser-assisted He^+^ milling. By optimizing pulsed laser power density, frequency, and pulse width, we reduce the carbonaceous byproducts and mill graphene gaps down to sub 10 nm in highly complex kiragami patterns.

## 1. Introduction

Graphene continues to attract attention as a material with intriguing physical and chemical properties and wide potential in chemical as well as biological sensors, energy conversion and storage, nanoelectronics, light-weight composite materials, and superconducting devices [1,2,3,4,5,6,7,8]. Graphene nanopatterning, also known as graphene kirigami/origami, has been intensively pursued in order to fabricate complex architectures [5,6,9,10,11,12,13,14,15]. Three-dimensional (3D) nanostructured graphene was investigated years ago, but a precise controlled patterning was not achieved until Blees et al. recently performed micrometer level kirigami via photolithography and plasma etching, and realized foldable 3D structures [16,17]. Attempts to enhance the patterning resolution have been pursued by ion milling [18,19,20], however, carbon contamination from the graphene transfer and patterning process, made it difficult to reproducibly push the resolution to the nanometer level. Additionally, carbon deposition in the beam interaction region due to cracking of the adsorbed hydrocarbon species or surface diffusing carbon complicates this issue [21,22]. Annealing samples in an Ar environment have been shown to reduce the carbon contamination [18,23,24], however, this was insufficient to completely remove residual carbon contaminants, and thus complementary approaches are necessary to ensure the reproducibility of the high-resolution patterning.

We have explored laser-assisted focused ion and electron beam induced processing using a synchronized pulsed laser to enhance the purity of deposits [25,26,27,28], mitigate subsurface ion beam damage [29], and enhance chemically assisted etching [30,31]. In this study, a systematic study of laser-assisted He^+^ milling of monolayer graphene is demonstrated. By tuning the parameters of the in situ pulsed laser beam, we mitigate competing carbon deposition and mill, as well as cut suspended graphene by the He^+^ beam. Direct-write kirigami patterns were realized with sub 10 nm resolution, enabling complex nanoscale graphene fabrication. Coupled with recent developments in the area of atom-by-atom fabrication using a scanning transmission electron microscope (STEM) and conventional semiconductor manufacturing techniques, these results may suggest a pathway toward device fabrication spanning from a macro- to nanoscale and atomic scale [32,33].

## 2. Materials and Methods

Commercial grade monolayer graphene samples on holey Si_3_N_4_ TEM membranes were purchased from Ted Pella (Redding, CA, USA). He^+^ milling was performed with a Zeiss ORION NanoFab He/Ne ion microscope (Carl Zeiss, White Plains, NY, USA). An accelerating voltage of 25 kV was used for all exposures. Beam currents were controlled from 1 to 3 pA. All patterns in this study were exposed with a constant pixel spacing of 0.25 nm and the linear dose from 1 × 10^4^ to 5 × 10^5^ ions/nm; the He^+^ beam pixel dwell time is a function of total dose, current, and the number of passes. Patterns were generated using Fibics NPVE pattern generating software and hardware scan controller. The laser system used is a prototype laser delivery system developed by Waviks, Inc. (Dallas, TX, USA). It has a 100 μm spot size and 915 nm wavelength. During the experiment the laser condition was controlled with the forward current ranging from 100 mA to 3 A, the frequency ranging from 10 to 3000 Hz, the pulse width from 1 µs to 1 ms, and the duty cycles from 0.01% to 3%. Detailed laser system information can be found in previous reports [31]. Finite element method (FEM) simulation was conducted and detailed descriptions can be found in the Supplementary Materials. STEM characterization was conducted using a Nion Ultra STEM US200 (Kirkland, WA, USA) operating at an accelerating voltage of 60 kV with a nominal beam current of 20 pA. Graphene samples used in the STEM study were prepared in house. Chemical vapor deposition was used to grow graphene on Cu foil. The surface was then coated with poly(methyl methacrylate) (PMMA) and the Cu foil was etched away in a bath of ammonium persulfate. The PMMA/graphene stack was rinsed in deionized water and transferred to a TEM grid before removal of the PMMA with acetone. Residual hydrocarbon contaminants were removed by baking in an Ar/O_2_ environment at 500 °C for 1.5 h [34].

## 3. Results and Discussions

**Figure 1:**
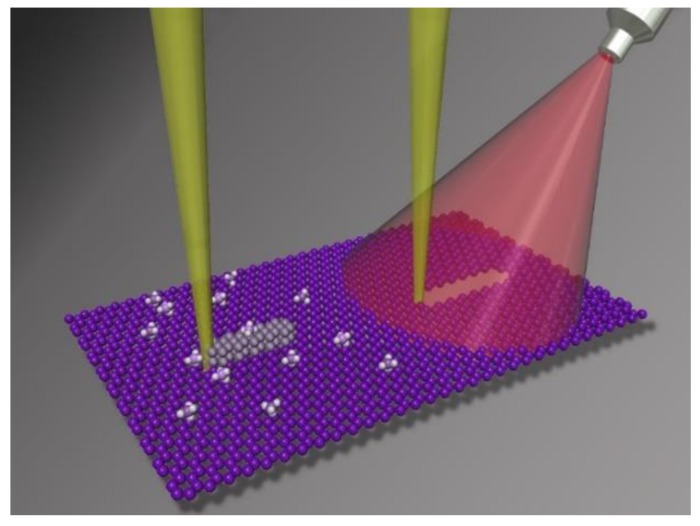
Schematic of He^+^ beam exposure on a single layer graphene sample. Carbon contamination introduced during transfer process impedes a clean cut by the He^+^ beam, and results in carbon deposition due to the cracking of residual hydrocarbon species. The carbon contamination can be reduced with the assist of an in situ pulsed laser, and thus ion milling is possible with appropriate laser conditions.

Figure 1 illustrates typical conditions for graphene He^+^ milling with and without in situ laser assist. Due to residual carbon contamination on the graphene samples, He^+^ beam exposure can produce competitive carbon deposition from the cracking of the hydrocarbon species adsorbed or surface diffused into the beam interaction region. In situ pulsed laser irradiation aids to reduce the carbon concentration, which enables the high precision nanoscale milling of suspended graphene.

**Figure 2:**
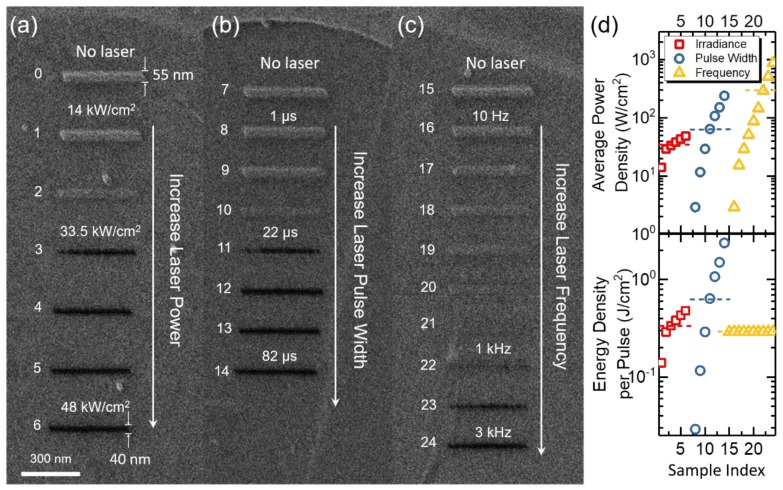
Tuning the laser condition via (**a**) irradiance, (**b**) pulse width, and (**c**) frequency to realize the transition between carbon deposition and He^+^ milling of monolayer graphene. Constant linear He^+^ beam scans with the length of 400 nm and the dose of 2 × 10^5^ ions/nm were used. The typical width of the carbon deposit and cut lines are 55 nm and 40 nm, respectively. The laser pulse width and frequency were set at 10 µs and 100 Hz, respectively, for the irradiance test shown in (**a**); the irradiance and frequency were set at 29 kW/cm^2^ and 100 Hz, respectively, for the pulse width test in (**b**); the irradiance and pulse width were set at 29 kW/cm^2^ and 10 µs, respectively, for the frequency test in (**c**); and (**d**) plots the average laser power density and energy density per pulse. Dashed lines mark the threshold values between depositions and mills.

Figure 2 shows the effects of different pulsed laser conditions such as laser irradiance (power density), pulse width, and frequency when writing 400 nm lines with He^+^. In this series of tests, the He^+^ dose was set at 2 × 10^5^ ions/nm with a 0.25 nm pixel spacing, and the time between He^+^ scans in this test was at least 5 minutes, which was, as demonstrated below, adequate to re-establish the pseudo-equilibrium conditions (see Supplementary Materials, Figure S1 for variable He^+^ dose). Figure 2a shows the results as a function of laser irradiance with fixed 10 μs pulse width and 100 Hz frequency. The bright line on top is the carbon deposition without the laser exposure. As the laser irradiance increases, less carbon deposition occurs and ion milling of the graphene proceeds at 33.5 kW/cm^2^ (Line 3 in Figure 2a). Clean line cuts can be achieved with ≥38 kW/cm^2^ laser irradiance (Line 4 to 6), where the width of milled lines is approximately 40 nm. Figure 2b and c shows the transition from deposition to milling by increasing the laser pulse width and pulse frequency, relative to a base laser condition of 29 kW/cm^2^, 10 μs, and 100 Hz. The approximate threshold pulse width and frequency to induce the milling upon the base condition are 22 μs and 500 Hz, respectively. The transition is particularly sensitive to the laser irradiance and pulse width, meanwhile the deposition and milling transition evolves less with the increase of frequency. The plot of the average pulsed laser power density in Figure 2d shows that the threshold average power density (marked as dash lines) is similar for the variable laser power and pulse width series, but the frequency test has a much higher threshold. The bottom plot in Figure 2d, is the energy density per pulse, which helps to clarify the trends. For the frequency study, the individual peak temperature at 29 kW/cm^2^ and 10 μs is below the threshold temperature, however, at higher frequencies the refresh time is short and at 1750 Hz is below the room temperature equilibration time, and thus the temperature rises and eventually exceeds the threshold temperature for the carbon desorption and diffusion to occur. The detailed laser condition for all the exposures are listed in Table 1.

**Figure 3:**
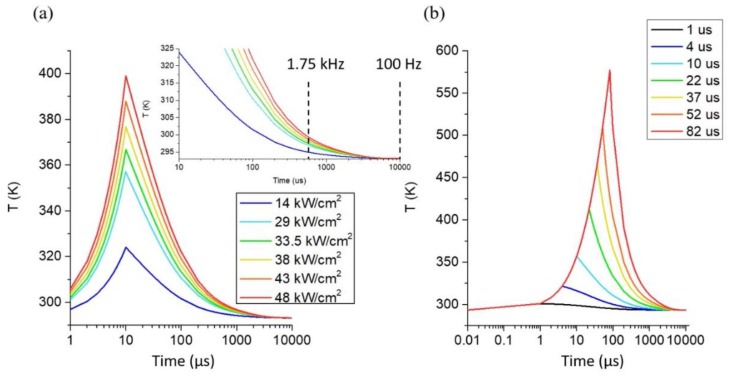
FEM simulation results for laser power (**a**) and pulse width (**b**) tests. The laser power profile shown in (**a**) is based on 10 μs pulse width and the pulse width profile shown in (**b**) is based on 29 kW/cm^2^ laser power density. The laser power is calculated by 90% irradiance over a laser spot area with a diameter of 100 μm (see the FEM simulation part for details). The mill happens with the power of 33.5 kW/cm^2^ and above in (**a**), and with the pulse width of 22 µs and above in (**b**), corresponding to a threshold peak temperature range of 366 K to approximately 411 K.

To further understand the underlining mechanisms, FEM simulation was conducted, and the result qualitatively approximates the actual configuration used in real experiments (a detailed description of FEM can be found in Supplementary Materials). Figure 3 illustrates the expected increase in temperature with increasing power and pulse width. Furthermore, it illustrates that at 100 Hz frequency, the thermal decay has an adequate time (~10 ms) to cool back to room temperature after each laser pulse. Thus, for 100 Hz, greater than ~200 mJ/cm^2^ per pulse energy density is required to raise the temperature high enough to desorb and diffuse the carbonaceous species from the laser region of interest. Notably, at short dwell times, the peak temperature scales with both laser power density and pulse width. At a higher frequency (>1 kHz), the thermal decay is incomplete, and the temperature does not cool to room temperature between pulses, which results in an increasing peak temperature as the number of pulses continues. Thus, while the initial pulse is below the temperature threshold to assist the focused ion beam milling, the steady-state peak temperature after many pulses clearly exceeds this threshold at ~1750 Hz frequency. The FEM simulation results provide rational for the observed trends and, in particular, the higher frequency threshold, although the absolute temperatures are uncertain.

As shown above, the pulsed laser assist can suppress the carbon deposition and enable ion milling. We attribute the suppression of carbon deposition to photothermal desorption of contaminants in the beam interaction region. Thus, for each parameter in Figure 2, a thermal threshold is reached in which the steady state carbon concentration is reduced sufficiently to allow the graphene to be milled.

**Figure 4:**
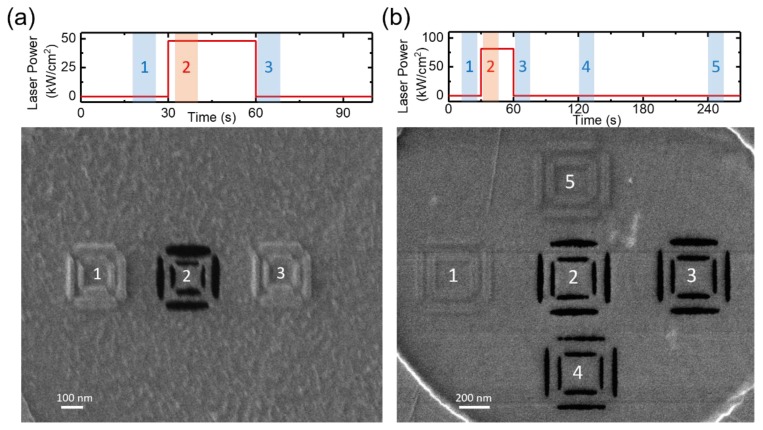
Effect of post-exposure time pulse laser-assisted graphene milling with low (**a**) and high (**b**) laser irradiance. Graphene milling versus carbon deposition switches immediately at 48 kW/cm^2^ laser irradiance. By increasing the irradiance to 81 kW/cm^2^, the residual carbon is mitigated for over 1 min after the laser is turned off. In this test the writing time for each pattern is 8 to 15 seconds. The *in situ* laser parameters were set at 10 μs pulse width and 100 Hz frequency and the He^+^ dosage was at 2 × 10^5^ ions/nm for all patterns.

Furthermore, a test was conducted to see if the photothermal treatment can permanently clean the sample for ex situ processing. Notably, the laser spot diameter is ~100 μm, thus, a relatively large area should be impacted. Figure 4a,b shows a comparison of the effect of a laser pretreatment on subsequent He^+^ exposures for two different laser irradiances of 48 and 81 kW/cm^2^, with the pulse width and frequency fixed at 10 μs and 100 Hz, respectively. In these tests, each pattern in Figure 4a took approximately 8 to 15 s with a 2 × 10^5^ ions/nm dose.

At 48 kW/cm^2^ (1 A) laser irradiance, an immediate switch between milling and deposition is realized, as shown in Figure 4a. Pattern 1 was exposed without the laser, which led to carbon deposition and pattern 2 was exposed with the in situ laser assist and milling was observed. Then, the laser was turned off and pattern 3 was exposed immediately, and deposition was observed again. When applying the laser assist at a higher irradiance of 81 kW/cm^2^, there was a latency time of over 1 minute in which milling was still possible. In Figure 4b, the first three patterns were completed following the same procedure as in Figure 4a, illustrating that the milling occurred after the laser was turned off. Pattern 4 was exposed ~1 minute after the laser was turned off–the milling still occurred, although the mill quality decreased at the edges. Finally, Pattern 5 was exposed three minutes after the laser was turned off and carbon deposition was again observed. The laser-on time for both tests was fixed at 30 seconds. Although it is not clear whether the carbon source was from the vapor phase, surface diffusion, or a combination of the two, an estimate of the surface diffusion coefficient was made by setting the diffusion distance, x, equal to the laser radius and solving for the diffusion coefficient (D), (i.e., $x=\sqrt{4Dt}$), where *t* = time (180 s); a value of ~3 × 10^−8^ cm^2^/s was obtained which is reasonable for large molecular weight hydrocarbons [35].

**Figure 5:**
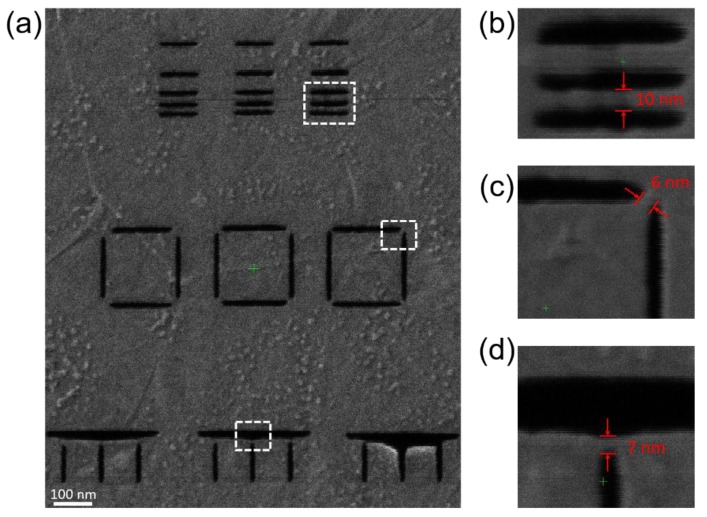
A series of patterns were made to explore of the highest milling resolution (**a**) and the patterning resolution with parallel (**b**), corner (**c**) and vertical (**d**) cuts. The width of the graphene nanobridges in these ions milled features can reach 10 nm in parallel cuts and approximately 6~7 nm in corner and vertical cuts.

**Figure 6:**
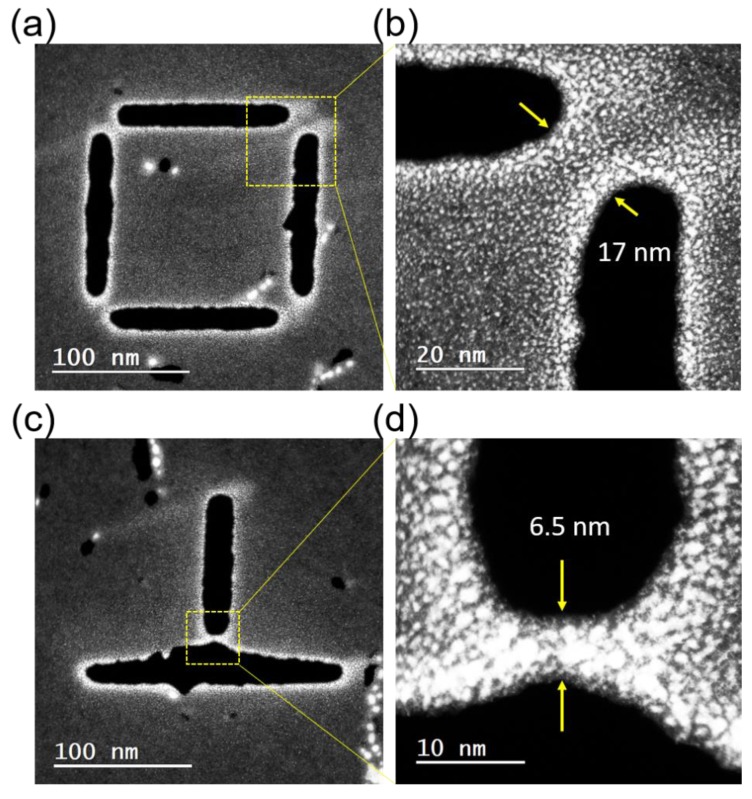
STEM images for preannealed graphene samples, after laser-assisted He^+^ milling. Images of a corner cut and a vertical cut are shown in (**a**,**b**) and (**c**,**d**), respectively. Carbon redeposition is clearly observed near the milled region.

Subsequent to demonstrating the enhanced milling via the laser assist, results of pattern resolution testing are demonstrated in Figure 5. The laser was set to 48 kW/cm^2^, 10 μs pulse width and 100 Hz, and the He^+^ linear dose was 2 × 10^5^ ions/nm. Figure 5a shows an overview of a series of patterns comparing parallel cuts, corner cuts, and vertical cuts. Figure 5b–d is high magnification images indicated by the white dashed squares in Figure 5a. The highest resolution of the graphene nanobridges created by ion milling reached 10 nm for parallel cuts and approximately 6~7 nm in corner and vertical cuts. The width of the milled gaps varied from 15 nm to 50 nm, which was due to the deformation of graphene around the milled areas. This deformation occurs during where the entire area was exposed to a low dose of the He^+^ beam. Figure S2 compares the feature before and after zoomed-in He^+^ imaging. It is observed that the patterned features within imaged areas, corresponding to Figure 5b–d, are seriously deformed. This may be due to the carbon redeposition induced by imaging. Features in other areas remained stable. Complementary STEM images of a pattern are shown in Figure 6. Although lattice imaging was attempted in order to reveal any peripheral graphene damage, as has been observed in other two-dimensional materials [26,30,31], the residual carbon around the deposits made it impossible to directly image the lattice at an atomic resolution. Images for a corner cut are shown in Figure 6a,b and images for a vertical cut are shown in Figure 6c,d. The residual carbon is observed even though the samples prepared for the STEM study were preannealed before the milling (see Experimental section for details), which indicates the carbon was redeposited near the edge of the mill. Although STEM did not reveal the level of damage to the patterned area, the contrast shown in the He^+^ microscope may suggest that the nanobridges are conductive and the lattice damage is minimal, as the previous work has shown that damage to the lattice will cause depletion of electrons to the pattern when imaging with positive ions [36].

**Figure 7:**
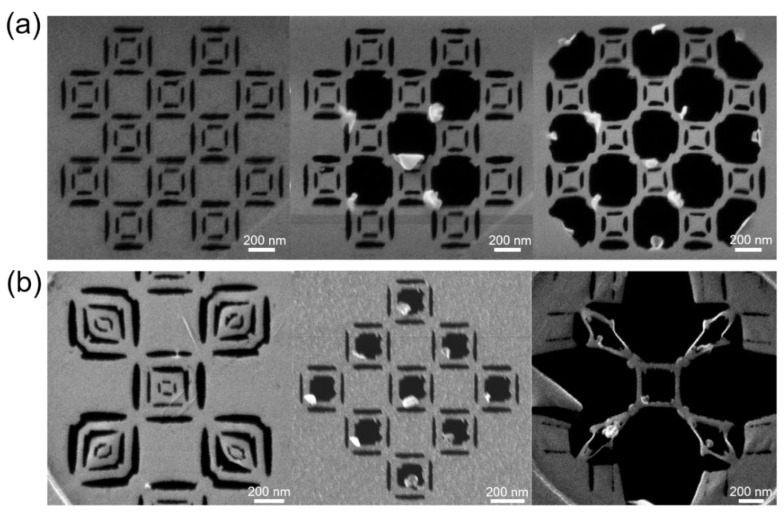
Complex features patterned under base parameters. (**a**) demonstrates a progressive milling procedure for a kirigami pattern and (**b**) shows several other compete patterns. With designed CAD files, these nanoscale graphene kirigami patterns can be easily realized by laser-assisted He^+^ milling. The suspended structures are stable with these graphene bridges with the width of tens of nanometers. Note in the right panel of (**b**) we intentionally induced strains in the suspended pattern and detailed steps of milling process can be found in Figure S3.

We have shown laser-assisted ion milling with sub 10 nm resolution, which enabled complex features to be written on these monolayer graphene samples. Figure 7 shows several examples of complex features patterned with the 48 kW/cm^2^ irradiance (1 A), 10 μs pulse width, and 100 Hz laser conditions, and a He^+^ linear dose of 2 × 10^5^ ions/nm. Figure 7a demonstrates a progressive milling process for a predesigned pattern, and more completed patterns are shown in Figure 7b. It is observed in these complex features that part of the graphene film was folded and rolled up during the milling process which resulted in the bright contrast in the images. Notably, in the last pattern shown in the right panel of Figure 7b, strain was intentionally introduced in the suspended nanobridges. Detailed patterning steps for this strained feature can be found in Supplementary Materials (Figure S3). These complex patterns are synthesized via a series of He^+^ cuts similar to macro kirigami. The suspended structures can remain stable, held by the graphene bridges with widths from 20 nm to 50 nm. This opens the possibility to direct write complex nanodevices on suspended membranes without traditional lithography.

## 4. Conclusions

In summary, we have shown a systematic study on laser-assisted He^+^ milling on monolayer graphene samples. By in situ pulsed laser, carbon contamination in commercial grade graphene samples can be effectively mitigated which enables ion milling. The detailed parameters of laser and He^+^ beam were carefully investigated with the demonstrated patterning resolution reaching sub 10 nm. Complex nanoscale kirigami patterns were then made with the suspended structure held by nanobridges. Heavy carbon redeposition is observed by STEM near the He^+^ milled area. This technique enables complex features to be direct written on suspended membranes, opening the gate to a variety of applications such as fabricating heterostructure carbon-based devices.

## Figures and Tables

**Table 1:** Detailed laser conditions of the three tests shown in Figure 2. The base parameter was set at 29 kW/cm^2^, 10 µs, and 100 Hz. Three exposure parameter series were conducted: laser power density, pulse width, and frequency.

Line ID	**0**	**1**	**2**	**3**	**4**	**5**	**6**			
**Irradiance (kW/cm2)**	N/A	14	29	33.5	38	43	48			
**Line ID**	**7**	**8**	**9**	**10**	**11**	**12**	**13**	**14**		
**Pulse Width (μs)**	N/A	1	4	10	22	37	52	82		
**Line ID**	**15**	**16**	**17**	**18**	**19**	**20**	**21**	**22**	**23**	**24**
**Frequency (Hz)**	N/A	10	52.5	100	175	300	500	1000	1750	3000

## Supplementary Materials

The following are available online at https://www.mdpi.com/2079-4991/9/10/1394/s1, Figure S1: He^+^ condition profile for graphene milling with laser; Figure S2: FEM simulation result for laser power (**a**) and pulse width (**b**) tests; Figure S3: Feature deformation due to the He^+^ exposure; detailed description of FEM simulation.
