# Addendum: Zhang, C., *et al.* Pulsed Laser-Assisted Helium Ion Nanomachining of Monolayer Graphene—Direct-Write Kirigami Patterns. *Nanomaterials* 2019, *9*, 1394

**DOI:** 10.3390/nano10020273

**Published:** 2020-02-06

**Authors:** Cheng Zhang, Ondrej Dyck, David A. Garfinkel, Michael G. Stanford, Alex A. Belianinov, Jason D. Fowlkes, Stephen Jesse, Philip D. Rack

**Affiliations:** 1 Department of Materials Science and Engineering, University of Tennessee, Knoxville, TN 37996, USA; czhang68@utk.edu (C.Z.); dgarfink@vols.utk.edu (D.A.G.); mstanfo3@gmail.com (M.G.S.); 2 Center for Nanophase Materials Sciences, Oak Ridge National Laboratory, Oak Ridge, TN 37831, USA; dyckoe@ornl.gov (O.D.); belianinova@ornl.gov (A.A.B.); fowlkesjd@ornl.gov (J.D.F.); sjesse@ornl.gov (S.J.)

The authors wish to make the following corrections to this paper [1]:

The funding section needs to be corrected. The updated information is included below:

**Funding:** The authors (P.D.R., J.D.F. and A.A.B.) acknowledge support from the Center for Nanophase Materials Sciences, which is a DOE Office of the Science User Facility. The authors (O.D. and S.J.) acknowledge support by the U.S. Department of Energy, Office of Science, Basic Energy Sciences, Materials Science and Engineering Division. C.Z. and M.G.S. acknowledge support from the U.S. Department of Energy (DOE) under grant no. DE-SC0002136. D.A.G. acknowledges the support by NSF CBET-1603780.

This addendum does not cause any changes to the results or conclusions in the original published paper.

The authors would like to apologize for any inconvenience caused to the readers by these changes.
