# Supplementary material for: Pulsed Laser-Assisted Helium Ion Nanomachining of Monolayer Graphene—Direct-Write Kirigami Patterns
Source: Nanomaterials (Basel). 2019 Sep 30;9(10):1394. doi: 10.3390/nano9101394 (PMC6835536; doi:10.3390/nano9101394)
Supplement: Supplementary file 1 [file nanomaterials-09-01394-s001.pdf]

nanomaterials

Article

Pulsed Laser Assisted Helium Ion Nanomachining of Monolayer Graphene—Direct Write Kirigami

Cheng Zhang[1,2], Ondrej Dyck[2], David A. Garfinkel[1], Michael G. Stanford[1], Alex A. Belianinov[2], Jason D. Fowlkes[2], Stephen Jesse[2], and Philip D. Rack[1,2]

[1] Department of Materials Science and Engineering, University of Tennessee, Knoxville, Tennessee 37996, USA
[2] Center for Nanophase Materials Sciences, Oak Ridge National Laboratory, Oak Ridge, Tennessee 37831, USA
* Philip D. Rack: prack@utk.edu

Figure S1. He^+ condition profile for graphene milling with laser. Two sets of dosage dependent exposures were conducted with different dwell time and repeats – the upper set used less repeats with long dwell time per pixel and the lower set used more repeats with short dwell time per pixel. The result shows that for the same dosage less repeats lead to cleaner cuts. During this test the current of He^+ was at 1.4 pA and the pixel size was fixed at 0.5 nm.

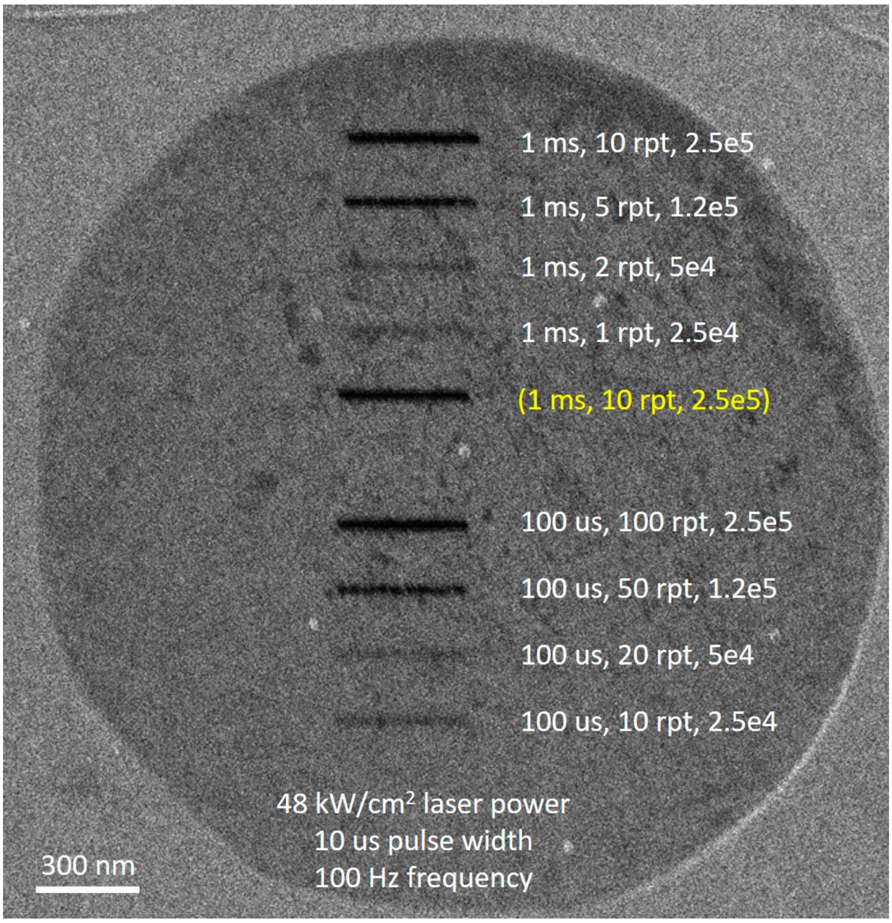

Figure S2. Feature deformation due to the He$^+$ exposure. Dashed squares indicates the higher magnification imaged areas corresponding to Fig 4b,c,d. The area that was imaged at high magnification clearly changes, while those not intensively imaged features in other areas remained stable.

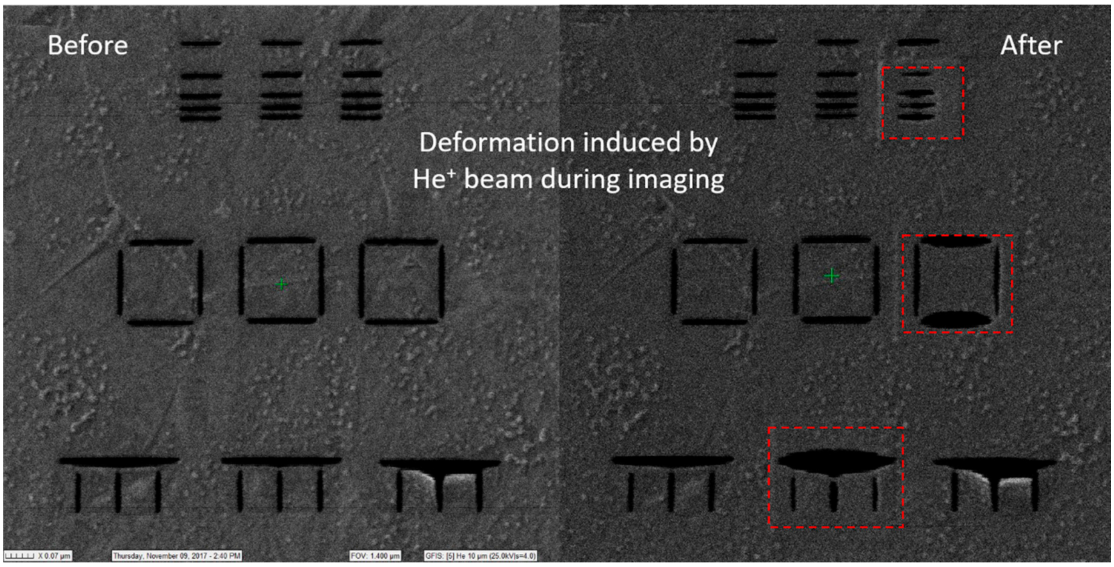

Figure S3. Steps to pattern the complex strained feature shown in Figure 6b right panel. The milling has to be processed in certain order to prevent the hallow part from collapsing.

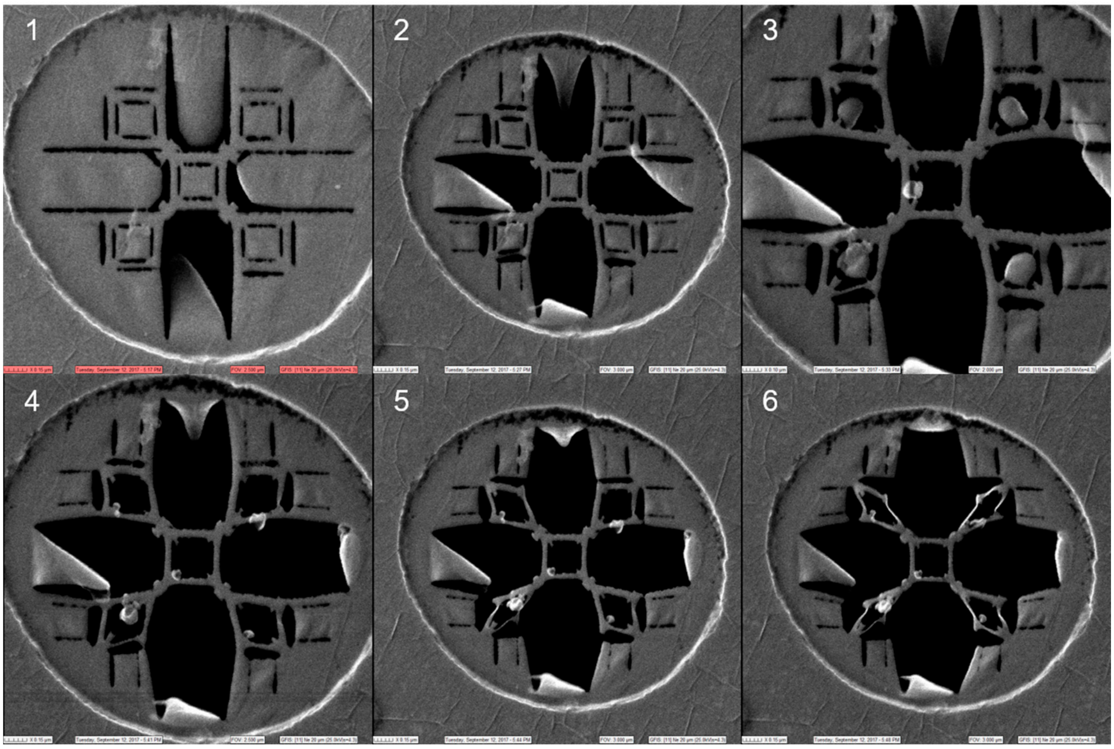

Finite Element Method Simulation Description

General Description

A model geometry was created to approximate the actual configuration used in real experiments. The model geometry is described with emphasis placed on the configuration differences between the experiments and simulations, as well as the justifications/assumptions supporting the model.

Real experiments were conducted on suspended single layer graphene (SLG) regions spanning a cylindrical micro–bore through the silicon nitride layer. These microbore regions were ignored in the FEM model, in favor of the use of a solid slab of silicon nitride to support the SLG, since (1) the total microbore volume is small relative to silicon nitride volume and (2) the laser beam radius is significantly larger that the radius of a single silicon nitride microbore.

Finite Element Method Simulation Parameters

3D Heat Equation

The 3D heat equation is solved using;

$$\rho C_p \frac{dT}{dt} = \nabla \cdot (k\nabla T) + Q(x,y,z) \;\; Q(x,y,z) = P(1-R)\frac{2\alpha}{\pi w^2} G(x,y) exp(-\alpha z)$$

The equation was solved on a suspended thin film domain. The terms α and R are the absorption coefficient and the reflectivity, respectively. G(x,y) is a gaussian laser irradiance profile with a full-width at 90 percent of maximum beam radius of w. The laser power is included as the term P. The width (x) and length (y) of the simulation domain were set at 1 mm x 1 mm. The SLG supported by silicon nitride was 0.5 mm x 0.5 mm. The remainder consisted of silicon nitride with 100 um thick silicon beneath. The thickness of the silicon nitride film (~z) was 200 nm. The heating source was applied at the top surface (z = 0 nm). The bottom surface of the silicon nitride was in contact with the vapor phase during real experiments, so the insulating boundary condition was applied at this interface. An initial simulation time step of 0.1 ns was used. The thermal conductivity (k) was assumed to be constant and will be discussed further below.

Laser beam size

The beam waist for the FEM was defined by setting the full–width at 90 percent of maximum, or FW90, equal to the experimentally observed beam diameter of 100 μm. The beam standard deviation (σ), a required parameter describing the Gaussian laser probe profile, is related to the FW90 by;

$$FW90 = 2\sqrt{2\ln 10}\,\sigma$$

so;

$$\sigma = \frac{100\ \mu m}{2\sqrt{2\ln 10}} = 23.3\ \mu m$$

Laser power

The laser power range investigated in the paper was;

$$P = 2.3 - 3.8\,W$$

Optical Index

The refractive index (n = 2.98) and extinction coefficient (k = 1.71) were derived from [1] The absorption coefficient is thus;

$$\alpha = \frac{4\pi k}{\lambda} = 2.3x10^{-2}\,nm^{-1}$$

The reflectivity used in the heating term was taken as the reflectivity of graphene;

$$R = \frac{(1-n)^2 + k^2}{(1+n)^2 + k^2} = 0.36$$

Please note, the potential influence of the reflectivity of the graphene/silicon nitride interface was neglected in the FEM.

Thermal Conductivity

The thermal conductivity (k) was taken as the defective value;

$$k = 400\frac{W}{m\,K}$$

according to [3] as a saturation value of k = 400 W/m/K has been determined for low energy electron irradiation at 20 keV [2] in the high defect limit.

SLG thickness

$$z_0 = 0.335x10^{-9}\,m$$

Physical properties

Reference [3] reports that the heat capacity and density for graphite are applicable for graphene;

$$c_p = 700\frac{J}{kg\,K}$$

$$\rho = 2000\,\frac{kg}{m^3}$$

Silicon nitride properties

$$c_p = 700\frac{J}{kg\,K}$$

$$\rho = 2000\,\frac{kg}{m^3}$$

$$k = 25\,\frac{W}{m\,K}$$

References

1. J. W. Weber, V. E. Calado and M. C. M. van de Sanden, "Optical constants of graphene measured by spectroscopic ellipsometry", *Appl. Phys. Lett.* **97**, 091904 (2010)
2. H. Malekpour and A. A. Balandin, "Raman–based technique for measuring thermal conductivity of graphene and related materials", *J. Raman. Spectrosc.* **49**, 106 (2018)

3. H. Cabrera, D. Mendoza, J. L. Benitez, C. Bautistia Flores, S. Alvarado and E. Marin, "Thermal diffusivity of few–layers graphene measured by an all–optical method", *J. Phys. D: Appl. Phys*. **48**, 465501 (2015)
